# Exploring the Prognosis of Breast Cancer with Synchronous Distant Nonregional Lymph Node Metastasis and Establishing a Predictive Model: A Population-Based Study

**DOI:** 10.1155/2022/5027457

**Published:** 2022-01-12

**Authors:** Hong Lin, Jianxiong Lin, Yanxuan Wu, Guoxi Liang, Jiating Sun, Liming Chen

**Affiliations:** ^1^Department of Medical Oncology, Cancer Hospital of Shantou University Medical College, Shantou, Guangdong, China; ^2^Department of Hematology and Oncology, Second Affiliated Hospital of Shantou University Medical College, Shantou, Guangdong, China; ^3^Department of Radiation Oncology, Cancer Hospital of Shantou University Medical College, Shantou, Guangdong, China; ^4^Department of Oncology, The First Affiliated Hospital of Shantou University Medical College, Shantou, Guangdong, China

## Abstract

**Background:**

We aimed to explore the prognosis of breast cancer patients with synchronous isolated distant-lymph node metastasis (SDLNM).

**Methods:**

We extracted information from the Surveillance, Epidemiology, and End Results Program. Kaplan-Meier and Cox regression analyses were used to compare overall survival (OS). Fine-Gray test was utilized to compare breast cancer-specific survival (BCSS). We applied propensity score matching (PSM) to balance confounders. In total, 692 SDLNM patients were allocated into training and validation cohorts. Univariate and multivariate analyses were implemented to determine independent prognostic variables. A nomogram predicting OS of SDLNM patients was constructed. Calibration curves and receiver operating characteristic curves were utilized to access the predictive model.

**Results:**

Cox regression and PSM analysis showed that the prognosis of SDLNM patients was similar to breast cancer patients in stage TnN3cM0 and superior to patients with other oligometastasis (SDLNM vs. TnN3cM0, *p* = 0.778; SDLNM vs. other oligometastasis: HR 0.767, 95% CI, 0.672-0.875, *p* < 0.001). A nomogram was established to predict 1-, 3-, and 5-year OS for SDLNM patients. All C-indexes and AUCs were greater than 0.7. Calibration curves implied accurate prediction. For patients receiving mastectomy, postoperative chemotherapy and radiotherapy were significant.

**Conclusions:**

Breast cancer with SDLNM has a similar OS and BCSS with locally advanced disease. Comprehensive treatment was associated with better prognosis compared with palliative therapy. We constructed a predictive model for SDLNM breast cancer. It will be necessary to design large-scale prospective trials to confirm our results and validate the predictive model.

## 1. Introduction

Breast cancer is the most common cancer among women. There were approximately 2 million new breast cancers worldwide in 2017, of which 1.9 million were women [1]. Approximately 5-8% of breast neoplasms are initially diagnosed as metastatic breast cancers (MBCs), which have a poor prognosis and are mainly treated with palliative therapy [2, 3]. Currently, according to the 8th Edition AJCC Cancer Staging, breast cancer with isolated distant lymph node metastasis belongs to MBC [4]. Regional lymph nodes of the breast include the ipsilateral axillary lymph node, ipsilateral internal breast lymph node, and ipsilateral supraclavicular lymph node, which are defined by the National Comprehensive Cancer Network [5]. Distant lymph nodes, including cervical, contralateral axillary, contralateral supraclavicular, and contralateral internal mammary lymph nodes, are nonregional lymph nodes. Staging and treatment for breast cancer patients with isolated distant lymph node metastasis still remain controversial. In fact, lymphatic drainage of the breast is dominated by axillary drainage, but external axillary drainage can also be found in 20-27% of cases, which includes the ipsilateral internal mammary chain (17%), intramammary (3%), interpectoral (2%), and supraclavicular (2%) nodes [6]. Contralateral axillary lymph node drainage is rare, only 0-2%, while reverse drainage of cervical nodes is even more infrequent [7, 8]. In general, damage to the ipsilateral lymphatic network increases the probability of abnormal drainage [7, 9–11]. Distant nonregional lymph node metastasis (DLNM) includes simultaneous and synchronous metastasis. The former indicates that metastasis existed at the initial diagnosis of breast cancer, and the latter indicates that DLNM took the form of recurrence after treatment. The two modalities are similar in proportion [12].

Supraclavicular lymph node metastasis (SLNM) is used to be considered as stage IV disease. However, Brito et al. [13] demonstrated that the survival outcome of SLNM after combined therapy was similar to that of stage N3b and was significantly superior to those with visceral metastasis. Thus, the American Joint Committee on Cancer (AJCC) staging system classified SLNM as stage III in 2002 [14]. Indeed, supraclavicular nodes belong to deep cervical nodes. Since tumor cells migrate to distant lymph nodes through lymph rather than blood, some investigators believe that DLNM should also not be classified as stage M1 [9, 11, 15–19]. Several studies propose that treatment for locally advanced breast cancer significantly improves the prognosis of DLNM patients [9, 10, 16–18, 20–22]. However, Guru et al. [23] considered that the prognosis of DLNM patients is similar to that of breast cancer patients with oligometastasis.

In order to address this controversial phenomenon, we conducted a study to compare the prognosis of synchronous isolated distant lymph node metastasis (SDLNM) with that of stage TnN3cM0 and other oligometastasis, as well as explore the role of multidisciplinary therapy for SDLNM patients. Moreover, we developed a predictive model to assess the prognosis of SDLNM patients. A nomogram is an alternative prognostic reference tool. It integrates complex demographic and clinicopathological features and translates them into visualized mathematical statistical model to achieve individualized prognostic prediction [24–26].

## 2. Materials and Methods

### 2.1. Material Acquisition

We retrieved materials from the Surveillance, Epidemiology, and End Results (SEER) program. The enrolled patients were required to meet the following criteria: (1) initially diagnosed with breast cancer, (2) were in stage TnN3cM0, or in stage IV with a specific oligometastasis site (including distant lymph node, distant soft tissue, bone, and viscera), (3) breast cancer was the first primary malignancy, (4) female and no more than 80 years of age, (5) survived for more than 1 month, and (6) diagnosed with breast cancer by histological methods. In addition, patients with bilateral breast cancer or unclear T stage, N stage, M stage, metastatic site, surgical mode, and molecular subtype in the database were excluded. All clinicopathologic information of the patients was registered at the initial diagnosis of breast cancer.

Demographic and clinicopathologic characteristics extracted from SEER were survival status, follow-up time, cause of death (cancer-specific event or not), age at diagnosis, race, marital status, histologica type, Scarff-Bloom-Richardson grading system (SBR grade), T stage, N stage, molecular subtype, metastatic site, and therapeutic experience.

In order to construct nomogram, age was transformed into a categorical variable. According to the TNM stage and metastatic site, we classified patients into 3 metastatic stages: (1) involvement of the distant lymph node (including cervical nodes, contralateral axillary, contralateral supraclavicular, and contralateral internal mammary nodes) in the absence of another metastatic site, (2) involvement of distant oligometastasis, (3) and patients in TnN3cM0 stage. The SEER database details the metastatic sites, including soft tissue, bone, and viscera. Distant oligometastasis meant that metastasis had been confirmed at only one site.

There was no personal identifying data appearing in our research. It was not necessary to apply for Institutional Review Board approval or get patient informed consent. Our study protocol was in agreement with the provisions of the Helsinki Declaration as revised in 2013.

### 2.2. Statistical Analyses

Overall survival (OS) duration was defined as the period from initial diagnosis of breast cancer to all-cause death. Breast cancer-specific survival (BCSS) duration referred to the period from initial diagnosis to cancer-specific death, while deaths from other causes were called competitive events.

A total of 9539 patients were enrolled in this study. Clinicopathological characteristics were compared using the chi-square test. Kaplan-Meier (KM) method and log-rank test were used to compare OS prognosis for patients at different stages. Cox univariate analysis was used to determine significant factors. Cox proportional hazards regression analysis with a forward stepwise procedure was used as a multivariate adjusted model to identify significant prognostic factors and evaluate the hazard ratios with 95% confidence intervals (95% CI). We also carried out Fine-Gray test to compare BCSS prognosis for patients at different stages [27, 28]. Propensity score matching (PSM) was adopted to modulate confounding factors between different populations. We performed logistic regression to evaluate variables associated with OS. Patients were matched on the basis of evaluated propensity using 1 : 1 matching via nearest method without replacement. A caliper of 0.05 was adopted.

Subsequently, patients were randomly allocated into a training and validation cohort at 6 : 4 ratio. In the training cohort, we utilized KM method and log-rank test to select significant variables. Then, the selected variables were entered into Cox multivariable survival analysis so as to obtain independent prognostic factors for OS prognosis. Based on these factors, a nomogram was developed to predict 1-, 3-, and 5-year OS of SDLNM patients. We performed internal and external validation in the training and validation cohort. Harrell's C statistic concordance index (C-index) and receiver operating characteristic (ROC) curves were used to evaluate discrimination of nomogram. Generally, the C-index and area under the ROC curve (AUC) range from 0.5 to 1.0, with 1.0 implying perfect coincidence and 0.5 implying complete randomness. Additionally, calibration plots for 1-, 3-, and 5-year survival were performed to estimate the accuracy of the model. High consistency between a predicted line and a 45-degree line indicates the accuracy of the model. Bootstrapping with 1,000 reiterations was adopted in these analyses. Additionally, independent prognostic factors for BCSS were screened by Fine-Gray univariate and multivariate analyses.

In univariate analysis, a two-sided *p* value < 0.10 was considered statistically significant, whereas a *p* value < 0.05 was statistically significant in other conditions. Statistical analyses were performed using SPSS (version 23.0) and R (version 3.6.3).

## 3. Results

### 3.1. Baseline Characteristics of the Included Patients

**Figure 1:**
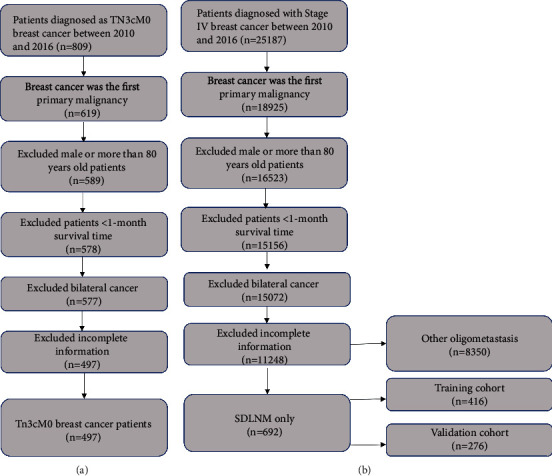
Flowchart of patient selection, (a) for TnN3cM0 stage, (b) for metastatic breast cancer with oligometastasis. SDLNM: synchronous isolated distant lymph node metastasis.

9539 patients with breast cancer from 2010 to 2016 were enrolled in our study (Figure 1). The median age was 58 (IQR 49-66) years old, and the median follow-up was 33 (IQR 16-45) months. The number of breast cancer patients with stage TnN3cM0, SDLNM, and other oligometastasis was 497, 692, and 8350, respectively. Patients with TNBC subtype had the highest mortality, while patients with other 3 subtypes had adjacent mortality, among which luminal B was correlated with the lowest mortality. All-cause survival probability in 1 year, 3 years, and 5 years for SDLNM patients was 86.5%, 59.9%, and 48.6%, respectively, while that for patients in stage TnN3cM0 was 92%, 65.2%, and 51.5%, respectively. As for patients with other oligometastasis, the OS was 85.8%, 57.8%, and 36.8% in 1 year, 3 years, and 5 years, respectively. Baseline features of the patients are summarized in Table 1.

### 3.2. Comparison of Diverse Metastatic Stages

**Figure 2:**
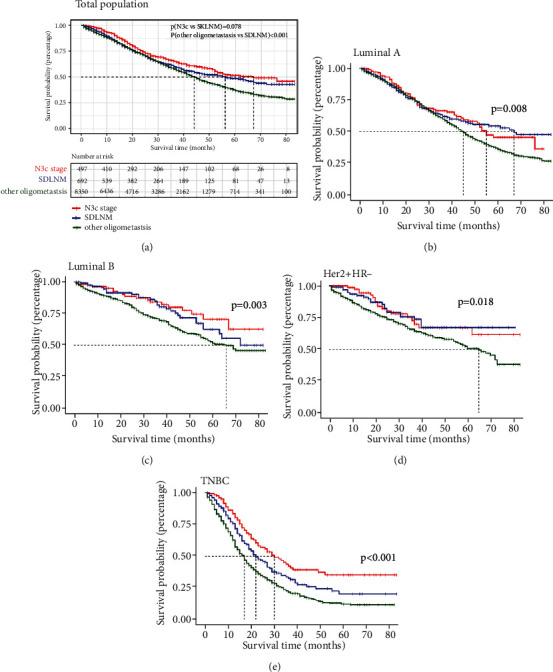
KM curves for patients with diverse stages in different populations ((a) for all included patients; (b) luminal A subtype; (c) luminal B subtype; (d) Her2 + HR- subtype; (e) TNBC subtype). SDLNM: synchronous isolated distant lymph node metastasis; Her2: human epidermal growth factor receptor type 2; HR: hormone receptor; TNBC: triple-negative breast cancer.

KM (Figure 2(a)) and cumulative incidence function (CIF, (Figure S1a)) curves demonstrated that the OS and BCSS of SDLNM patients were similar to that of patients in stage TnN3cM0 and were both superior to that of MBC patients with other oligometastasis. Figures 2(b)–2(d) and Figure S1 (b-d) show that for luminal A, luminal B, and Her2 + HR- molecular subtypes, the abovementioned conclusion held true. However, for TNBC subtype, patients in stage TnN3cM0 were associated with better OS and BCSS prognosis compared with patients in other 2 groups, whereas patients with SDLNM had longer survival compared with patients with other oligometastasis (Figure 2(e) and Figure S1e). Cox univariate analysis verified that age, marriage, histology, SBR grade, T stage, metastatic type, subtype, surgery, chemotherapy, and radiotherapy were significant factors for OS. After adjusting for other prognostic parameters via using a Cox stepwise regression model, involvement of SDLNM was associated with similar OS and BCSS prognosis compared with patients in the TnN3cM0 stage (OS: *p* = 0.778; BCSS: *p* = 0.670) and had a greater survival advantage than other MBC patients (OS: hazard ratio, 0.767, 95% CI, 0.672-0.875, *p* < 0.001; BCSS: hazard ratio, 0.755, 95% CI, 0.652-0.874, *p* < 0.001).

### 3.3. Propensity Score Matching Results

**Figure 3:**
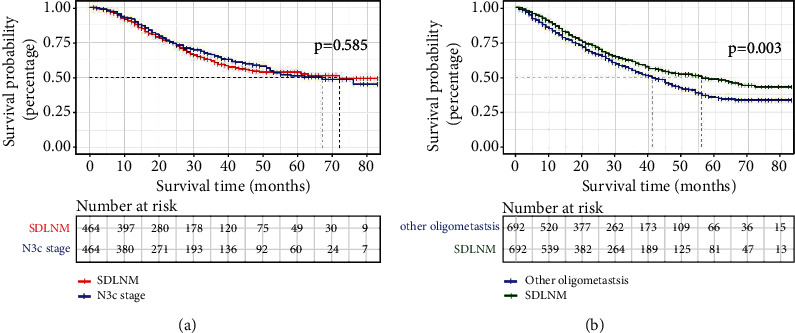
KM curves for PSM cohorts. (a) PSM cohort 1 (SDLNM vs. TnN3cM0 stage); (b) PSM cohort 2 (SDLNM vs. other oligometastasis). PSM: propensity score matching; SDLNM: synchronous isolated distant lymph node metastasis.

Since there were imbalance characteristics between diverse metastatic stages, we performed PSM to obtain 2 matched cohorts that could be used to further verify the large survival difference. There were 928 patients in matched cohort 1 (464 SDLNM patients and 464 stage TnN3cM0 patients) and 1384 patients in matched cohort 2 (692 SDLNM patients and 692 other oligometastatic patients). The baseline features of these two cohorts are illustrated in Table S1 and Table S2. In matched cohort 1, no significant difference was found between SDLNM patients and stage TnN3cM0 patients. Therefore, stage TnN3cM0 and SDLNM breast cancer patients still had similar prognosis (Figure 3(a) and Figure S2a) after PSM. In matched cohort 2, SDLNM patients had longer OS and BCSS than patients with other oligometastasis (Figure 3(b) and Figure S2b). The PSM results further verified that SDLNM patients had survival identical to TnN3cM0 and were different from stage IV.

### 3.4. Descriptive Analysis of SDLNM Patients

**Figure 4:**
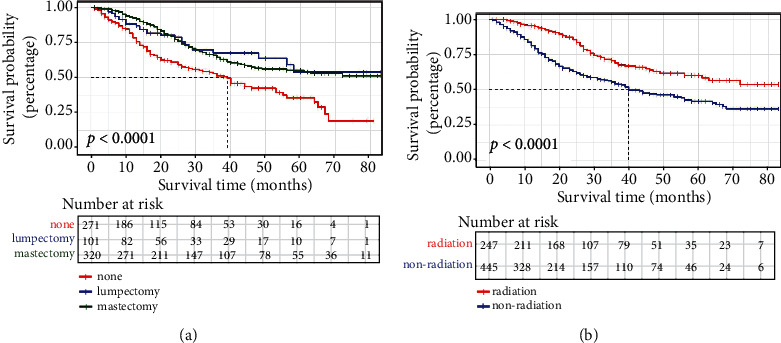
KM curves of surgery modes (a) and radiotherapy (b) for breast cancer patients with SDLNM. SDLNM: synchronous isolated distant lymph node metastasis.

Among the 692 patients with SDLNM, luminal A, luminal B, Her2 + HR-, and TNBC subtypes accounted for 41.62% (288/692), 18.35% (127/692), 14.31% (99/692), and 25.72% (178/692), respectively. TNBC was associated with the highest mortality (57.3%). Of note, SDLNM was associated with strong tumor aggressiveness. The proportion of SDLNM patients in stage T4, SBR grade 3, and stage N3 was 39.88% (276/692), 48.41% (335/692), and 40.75% (282/692), respectively. There were 628 (90.75%) patients who had positive ipsilateral nodes. As for curative therapy, 63.84% (421/692) patients underwent local surgery, among which 320 (46.24%) patients received mastectomy. 5-year OS for patients without surgery, with lumpectomy or with mastectomy was 35.7%, 53.9%, and 55.3%, respectively. Additionally, 578 (83.53%) and 247 (35.69%) patients suffered from chemotherapy and radiotherapy, respectively. After adjusting for other prognostic factors, mastectomy clearly improved OS prognosis of patients (hazard ratio, 0.668; 95% CI, 0.487-0.917, *p* = 0.012). However, lumpectomy did not bring survival prognosis benefit (*p* = 0.475). Furthermore, patients who did not receive radiotherapy were prone to have poor prognosis (hazard ratio, 1.745; 95% CI, 1.249-2.439, *p* = 0.001). Of importance, chemotherapy did not seem to have significant effect on OS for SDLNM patients.  Figure 4 shows the KM curves of patients receiving surgery and radiotherapy.

As mentioned earlier, the prognosis of SDLNM patients is quite different from other MBC patients. To precisely evaluate the prognosis for this patient population and assist clinical decision making, we aimed to develop predictive models by first randomly placing 416 and 276 patients into a training and validation cohort, respectively. The median follow-up duration in the training and validation cohorts was 32.5 (IQR 15.25-46) months and 33 (IQR 16.25-47.75) months, respectively. There were 152 (36.5%) death events in the training cohort, of which 137 (32.93%) were caused by cancer. Similarly, in the validation cohort, 97 (35.1%) and 92 (33.3%) patients suffered from all-cause and cancer-specific death, respectively. The baseline characteristics of SDLNM patients are shown in Table 2.

### 3.5. Predictive Model for SDLNM Patient Prognosis

Univariate analysis in the training cohort showed marital status, race, SBR grade, T stage, molecular subtype, surgery, and radiotherapy were associated with OS prognosis for breast cancer patients with SDLNM. Cox multivariate analysis showed that SBR grade, T stage, molecular subtype, surgery, and radiotherapy were independent prognostic parameters. The results of univariate and multivariate analyses are shown in Table 3. It is worth noting that N stage and chemotherapy were not significant predictors for OS.

**Figure 5:**
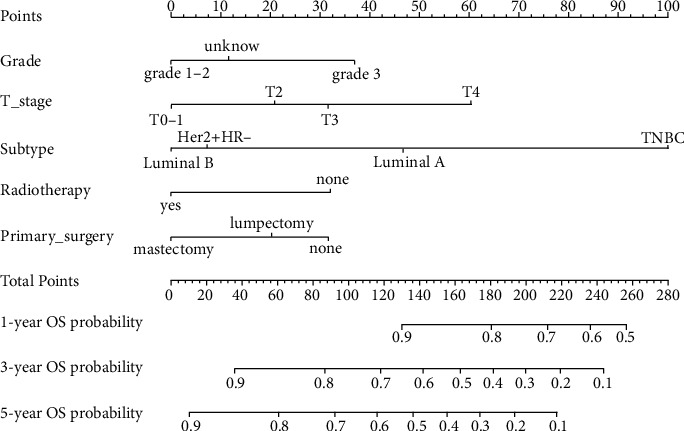
Nomogram to predict the 1-year, 3-year, and 5-year OS for breast cancer patients with SDLNM. OS: overall survival; Her2: human epidermal growth factor receptor type 2; HR: hormone receptor; TNBC: triple-negative breast cancer; SDLNM: synchronous isolated metastatic distant lymph node metastasis.

The abovementioned significant variables were integrated to establish a prediction model. A nomogram for predicting 1-, 3-, and 5-year OS was established (Figure 5(a)). Each predictor utilized to develop the nomogram was assigned a score. By adding all scores that relied on patient clinicopathological features to obtain a total score, a straight line from the “Points” to “OS probability” axis was drawn to estimate the prognosis of patients. Of importance, mastectomy brought significant OS advantages for SDLNM patients.

**Figure 6:**
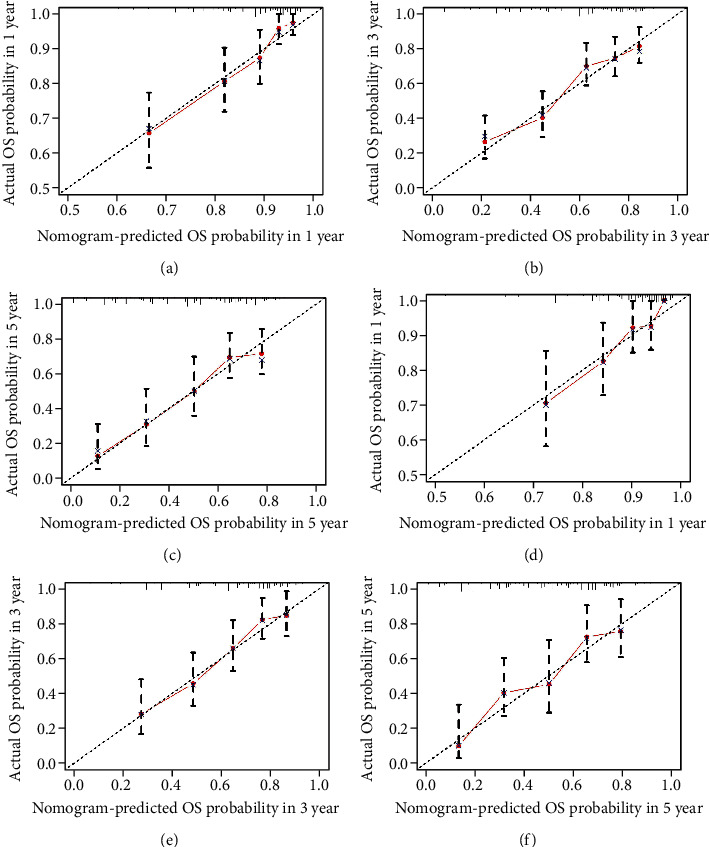
Internal and external calibration curves for nomogram predicting the 1-, 3-, and 5-year OS of breast cancer SDLNM patients. (a)–(c) Training cohort. (d)–(f) Validation cohort. OS: overall survival; SDLNM: synchronous isolated distant lymph node metastasis.

The C-index of the nomogram for predicting the 1-, 3-, and 5-year OS prognosis was 0.731 (95% CI 0.692-0.770) and 0.722 (95% CI 0.671-0.773) in the training and validation cohort, respectively. ROC curves are plotted in Figure S3. All C-indexes and AUCs were greater than 0.7, which implies good predictive discrimination. Calibration curves indicated the precise predictive efficiency of the models both in training and validation cohort (Figure 6).

Fine-Gray univariate and multivariate analysis revealed that SBR grade, T stage, molecular subtype, and radiotherapy were significantly associated with BCSS (Table 3).

### 3.6. Postmastectomy Chemotherapy and Radiotherapy Improve OS

**Figure 7:**
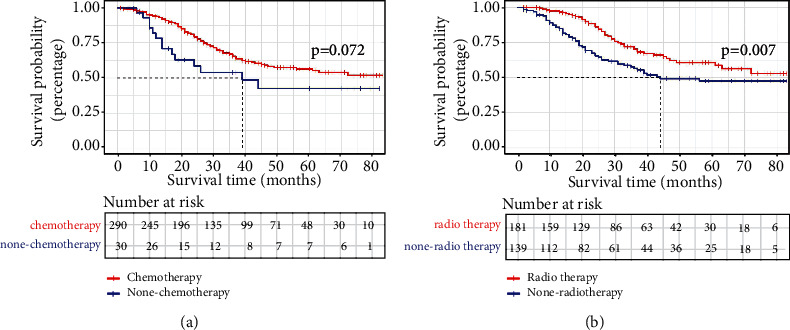
KM curves for chemotherapy (a) and postoperative radiotherapy (b) for SDLNM patients. SDLNM: synchronous isolated distant lymph node metastasis.

Because of the prognostic comparability between SDLNM and TnN3cM0 stage, we explored whether chemotherapy provided a survival advantage. There were 320 SDLNM patients who underwent mastectomy, and 101 patients received lumpectomy. KM analysis indicated that chemotherapy and radiotherapy increased OS of patients who underwent mastectomy (Figure 7), but not patients who received lumpectomy. After adjusting for other factors, patient who received mastectomy still benefitted from chemotherapy and postoperative radiotherapy (chemotherapy: hazard ratio, 1.973, 95% CI, 1.033-3.769, *p* = 0.040; postoperative radiotherapy: hazard ratio, 1.669, 95% CI, 1.102-2.529, *p* = 0.016).

## 4. Discussion

Breast cancer with SDLNM is an infrequent disease. Cervical lymph node metastasis (CLNM) occurs in only 1% of breast cancers, whereas the incidence of contralateral axillary node metastasis (CAM) is 1.9-6.0% ^6,19^. Perre et al. [29] applied regional lymphoscintigraphy to 23 patients before and after breast surgery and found that the lymphatic drainages of 7 postoperative patients drained to the contralateral lymphatic network, among which 6 drained to the axilla and 1 was diverted to the internal mammary nodes, implying that the incidence of contralateral internal mammary node metastasis may be much lower than that of CAM.

Prospective and retrospective studies evaluating the treatment and prognosis of breast cancer patients with DLNM are difficult to perform due to the extremely low incidence. Most relevant literature involves case reports [10, 11, 18, 21, 22, 30–32]. Because of the lack of large sample size in studies, staging and prognosis of DLNM patients in the absence of other distant metastasis still remain controversial. In terms of anatomy, tumor cells migrate to distant lymph nodes via lymphatic network rather than blood circulation, so DLNM should not be categorized as stage M1 [9]. Several reports indicate that the prognosis of breast cancer patients with cervical and contralateral node metastasis is similar to that of patients at the N3c stage [9, 11, 15–19]. Moossdorff et al. [17] summarized 24 previous studies and proposed that the survival of patients with contralateral lymph node metastasis is not comparable to patients with other distant metastatic diseases (the average OS after 50.3-month follow-up was 82.6%). Recently, an Italian study analyzed 47 patients with CAM and suggested that the estimated 5-year OS and progression-free survival after multidisciplinary treatment are 72% (95% CI 54-83) and 61% (95% CI 44-74), respectively [16]. In addition, another retrospective study KROG 18-02 reviewed 78 patients with cervical lymph node metastasis from 7 institutions and found that the 5-year OS, disease-free survival, locoregional relapse-free survival, and distant metastasis-free survival after treatment were 68.6%, 46.7%, 68.4%, and 57.0%, respectively, and quite different from the 5-year OS for MBC of 26-49% [15]. However, Guru et al. [23] reviewed 23 breast cancer patients with metachronous CAM and concluded that the prognosis of CAM patients was similar to that of MBC patients with oligometastasis. Our research substantiates that the OS and BCSS prognoses of SDLNM breast cancer patients are similar to those for N3c stage patients and are superior to those of other oligometastatic MBC patients, especially for long-term prognosis. For short-term prognosis, SDLNM has few survival advantages compared with the other 2 stages. It is worth mentioning that only approximately half the population in our study received surgery and radiation, and fewer received combination therapy, whereas all participants in the above studies received comprehensive treatment regimens.

Generally, lymph flows along interlobular vessels of the breast into the subareolar plexus and then follows the mammary veins to the axilla (75%). Lymph from the medial breast can also flow into the parasternal lymph nodes [33]. DLNM may be caused by the diversion and retrogradation of lymphatic drainage following the destruction of the ipsilateral lymphatic network [7, 9–11]. Associated risk factors include large mammary neoplasm, previous mammary or axillary surgery and radiation, and large tumor burdens in the ipsilateral axilla [7, 9, 11]. Allweis et al. [31] retrospectively analyzed 21 cases of CAM, among which 10 cases were synchronous and 11 were metachronous. For patients with internal breast involvement, a retrosternal route crossing to the contralateral was possible. Morcos et al. [12] retrospectively analyzed 21 cases of CAM, among which 10 cases were synchronous and 11 were metachronous. The histopathological features of CAM patients were significantly worse, such as lymphatic vascular invasion (81%), high histological grade (81% grade 3), large primary breast neoplasms (95% cT3/cT4), estrogen receptor negativity (52%), and overexpression of Her2 (42%). Our study is in agreement with this. Among 692 participants, patients with invasive characteristics including large tumors, high SBR grade, ipsilateral lymph node metastasis, and TNBC subtype accounted for a high proportion.

Clinicians generally adopt comprehensive treatment for DLNM patients and to obtain acceptable curative outcomes [9–11, 16–18, 20–22, 32]. Some clinicians also implement palliative treatment [19, 30]. Several retrospective studies suggest that surgery and systemic therapy enhance the prognosis of patients with DLNM [9, 15, 16]. However, there are some differences between operational methods and combined schemes. Kim et al. [15] considered that cervical lymph node metastasis patients could benefit from systemic chemotherapy and locoregional therapy for the ipsilateral breast, but neck dissection and radiotherapy does not improve locoregional relapse-free survival and disease-free survival. Magnoni et al. [16] found that contralateral axillary lymph node dissection provide a prognostic advantage for CAM patients. However, chemotherapy and postaxillary lymph node dissection radiotherapy did not appear to improve OS (*p* = 0.13 and *p* = 0.65) or disease-free survival (*p* = 0.25 and *p* = 0.5). In addition, a Chinese study reviewed 25 CAM cases and proposed that the combination of surgery, systemic chemotherapy, radiotherapy, and antihormone therapy is more effective in controlling disease compared with mastectomy and axillary lymph node dissection alone [20]. Oppositely, Wong et al. [19] performed a retrospective analysis of 15 synchronous CAM patients, and the comparison showed no significant difference in 5-year cancer-specific survival between the palliative and operative groups (68.6% vs. 80.0%, *p* = 0.79). Our research study suggests that mastectomy with chemotherapy and postoperative radiotherapy significantly improves OS for breast cancer patients with SDLNM, while lumpectomy, chemotherapy, or postlumpectomy chemotherapy does not increase OS. In brief, a comprehensive curative program on SDLNM breast cancer patients, just like that performed for locally advanced breast cancer patients, remarkably prolongs OS.

This is the first large-sample study that retrospectively analyses the prognosis and treatment of breast cancer patients with SDLNM, with the purpose of providing a reference for staging and curative planning. There are some limitations in our research. First, due to the limited treatment information from the SEER database, we were unable to analyze the roles of lymphadenectomy of metastatic lymph nodes, which will require further studies to explore and improve our models. Second, the prognosis of patients with SDLNM was disparate among diverse subtypes. Therefore, it is important to assess the prognosis and optimal treatment for SDLNM patients according to molecular subtypes in the future. Finally, our study only includes patients from the SEER, so a subsequent study from other countries will be needed for verification.

## 5. Conclusions

Breast cancer with SDLNM might be classified as locally advanced disease, but not metastatic disease. Comprehensive therapy combined with mastectomy, postchemotherapy, and postoperative radiotherapy brings significant survival advantage. We developed predictive models to evaluate the 1-, 3-, and 5-year OS for SDLNM patients. This optional tool may help clinicians formulate therapy and follow-up arrangement based on individual conditions and provide a reference for the design of subsequent prospective trials.

## Figures and Tables

**Table 1:** Demographic and clinicopathologic features of all included patients.

Characteristics	TnN3cM0 (%)	SDLNM (%)	Other oligometastasis (%)	*p* value
Age				<0.001
≤50 y	186 (37.4)	218 (31.5)	2371 (28.4)	
51-65 y	205 (41.2)	302 (43.6)	3631 (43.5)	
66-80 y	106 (21.3)	172 (24.9)	2348 (28.1)	
Race				<0.001
Caucasian	351 (70.6)	481 (69.5)	6364 (76.2)	
Black/AI	108 (21.7)	135 (19.5)	1371 (16.4)	
Asian	38 (7.6)	76 (11.0)	615 (7.4)	
Marriage				0.506
Married	256 (51.5)	343 (49.6)	4082 (48.9)	
Single and unknown	241 (48.5)	349 (50.4)	4268 (51.1)	
Histology				<0.001
IDC	402 (80.9)	556 (80.3)	6056 (72.5)	
Non-IDC	95 (19.1)	136 (19.7)	2294 (27.5)	
SBR grade				<0.001
Grade 3	255 (51.3)	335 (48.4)	2462 (29.5)	
Grade 1-2	126 (25.4)	184 (26.6)	3651 (43.7)	
Unknown	116 (23.3)	173 (25.0)	2237 (26.8)	
T stage				<0.001
T0-1	73 (14.7)	102 (14.7)	1247 (14.9)	
T2	163 (32.8)	207 (29.9)	3266 (39.1)	
T3	93 (18.7)	107 (15.5)	1607 (19.2)	
T4	168 (33.8)	276 (39.9)	2230 (26.7)	
Subtype				<0.001
Luminal A	174 (35.0)	288 (41.6)	5531 (66.2)	
Luminal B	113 (22.7)	127 (18.4)	1368 (16.4)	
Her2 + HR-	82 (16.5)	99 (14.3)	564 (6.8)	
TNBC	128 (25.8)	178 (25.7)	887 (10.6)	
Surgery				<0.001
None	95 (19.1)	271 (39.2)	4857 (58.2)	
Lumpectomy	87 (17.5)	101 (14.6)	1057 (12.7)	
Mastectomy	315 (63.4)	320 (46.2)	2436 (29.2)	
Radiotherapy				<0.001
Yes	293 (59.0)	247 (35.7)	2133 (25.5)	
No	204 (41.0)	445 (64.3)	6217 (74.5)	
Chemotherapy				<0.001
Yes	463 (93.2)	578 (83.5)	5013 (60.0)	
No	34 (6.8)	114 (16.5)	3337 (40.0)	

SDLNM: synchronous isolated distant lymph node metastasis; Her2: human epidermal growth factor receptor type 2; AI: American Indian; HR: hormone receptor; IDC: infiltrating duct carcinoma; SBR grade: Scarff-Bloom-Richardson grading system; TNBC: triple-negative breast cancer.

**Table 2:** Baseline characteristics of breast cancer patients with SDLNM.

Characteristic	Training cohort (*n* = 416)	Validation cohort (*n* = 276)	*p* value
Age (%)			0.570
≤50 y	136 (32.7)	82 (29.7)	
51-65 y	175 (42.1)	127 (46.0)	
66-80 y	105 (25.2)	67 (24.3)	
Race (%)			0.716
White	294 (70.7)	187 (67.8)	
Black/AI	78 (18.8)	57 (20.7)	
Asian	44 (10.6)	32 (11.6)	
Marriage (%)			0.555
Married	210 (50.5)	133 (48.2)	
Single and unknown	206 (49.5)	143 (51.8)	
Histology (%)			0.661
IDC	332 (79.8)	224 (81.2)	
Non-IDC	84 (20.2)	52 (18.8)	
SBR grade (%)			0.455
Grade 3	206 (49.5)	129 (46.7)	
Grade 1-2	113 (27.2)	71 (25.7)	
Unknown	97 (23.3)	76 (27.5)	
T stage (%)			0.477
T0-1	66 (15.9)	36 (13.0)	
T2	116 (27.9)	91 (33.0)	
T3	65 (15.6)	42 (15.2)	
T4	169 (40.6)	107 (38.8)	
N stage (%)			0.124
N0	47 (11.3)	17 (6.2)	
N1	158 (38.0)	108 (39.1)	
N2	44 (10.6)	36 (13.0)	
N3	167 (40.1)	115 (41.7)	
Subtype (%)			0.367
Luminal A	167 (40.1)	121 (43.8)	
Luminal B	74 (17.8)	53 (19.2)	
Her2 + HR-	58 (13.9)	41 (14.9)	
TNBC	117 (28.1)	61 (22.1)	
Surgery (%)			0.366
None	154 (37.0)	117 (42.4)	
Lumpectomy	63 (15.1)	38 (13.8)	
Mastectomy	199 (47.8)	121 (43.8)	
Radiotherapy (%)			0.937
Yes	148 (35.6)	99 (35.9)	
No	268 (64.4)	177 (64.1)	
Chemotherapy (%)			0.247
Yes	353 (84.9)	225 (81.5)	
No	63 (15.1)	51 (18.5)	
Status (%)			0.708
Alive	264 (63.5)	179 (64.9)	
Dead	152 (36.5)	97 (35.1)	

AI: American Indian; Her2: human epidermal growth factor receptor type 2; HR: hormone receptor; IDC: infiltrating duct carcinoma; SBR grade: Scarff-Bloom-Richardson grading system; SDLNM: synchronous isolated distant lymph node metastasis; TNBC: triple-negative breast cancer.

**Table 3:** Univariate and multivariate analyses for OS and BCSS of breast cancer patients with SDLNM.

Variables	OS			BCSS		
	Univariate	Multivariate		Univariate	Multivariate	
	*p*	Hazard ratio (95% CI)	*p*	*p*	Hazard ratio (95% CI)	*p*
Age	0.31			0.836		
Marriage	0.046	1.27 (0.91, 1.78)	0.156	0.132		
Race	0.025			0.023		
White		Reference			Reference	
Black/AI		1.24 (0.84, 1.84)	0.283		1.42 (0.93, 2.17)	0.100
Asian		0.59 (0.28, 1.23)	0.160		0.37 (0.32, 1.44)	0.310
Histology	0.24			0.557		
SBR grade	<0.001			<0.001		
Grade 3		Reference			Reference	
Grade 1-2		0.58 (0.37, 0.91)	0.016∗		0.52 (0.31, 0.86)	0.011∗
Unknown		0.69 (0.45, 1.05)	0.082		0.72 (0.45, 1.16)	0.180
T stage	0.013			0.094		
T4		Reference			Reference	
T3		0.65 (0.39, 1.10)	0.106		0.64 (0.38, 1.08)	0.097
T2		0.56 (0.37, 0.84)	0.005∗		0.62 (0.38, 0.98)	0.043∗
T0-1		0.41 (0.24, 0.69)	<0.001∗		0.53 (0.30, 0.95)	0.032∗
N stage	0.118			0.098		
N3					Reference	
N2					0.64 (0.38, 1.08)	0.057
N1					0.62 (0.38, 0.99)	0.580
N0					0.53 (0.30, 0.95)	0.530
Subtype	<0.001			<0.001		
Luminal A		Reference			Reference	
Luminal B		0.49 (0.29, 0.87)	0.014∗		0.43 (0.23, 0.80)	0.007∗
Her2 + HR-		0.56 (0.30, 1.04)	0.064		0.49 (0.25, 0.94)	0.032∗
TNBC		2.21 (1.49, 3.28)	<0.001∗		2.07 (1.31, 3.27)	0.002∗
Surgery	0.001			0.004		
None		Reference			Reference	
Lumpectomy		0.85 (0.49, 1.47)	0.555		1.01 (0.58, 1.77)	0.960
Mastectomy		0.63 (0.41, 0.95)	0.026∗		0.79 (0.50, 1.25)	0.310
Radiotherapy	<0.001	1.61 (1.07, 2.44)	0.024∗	<0.001	1.83 (1.19, 2.84)	0.006∗
Chemotherapy	0.171			0.848		

BCSS: breast cancer-specific survival; CI: confidence intervals; Her2: human epidermal growth factor receptor type 2; HR: hormone receptor; OS: overall survival; SBR grade: Scarff-Bloom-Richardson grading system; SDLNM: synchronous isolated distant lymph node metastasis; TNBC: triple-negative breast cancer.

## Data Availability

The data of this study are from SEER database.

## Supplementary Materials

Supplementary Materials Table S1: characteristics of BC patients with SLDNM or stage TnN3cM0 before and after PSM. Table S2: characteristics of BC patients with SLDNM or other oligometastasis before and after PSM. Figure S1: cumulative mortality curves for patients with diverse stages in different populations ((a) for all included patients; (b) for luminal A subtype; (c) for luminal B subtype; (d) for Her2 + HR- subtype; (e) for TNBC subtype). SDLNM: synchronous isolated distant lymph node metastasis; Her2: human epidermal growth factor receptor type 2; HR: hormone receptor; TNBC: triple-negative breast cancer; BCSM: breast cancer-specific mortality. Figure S2: cumulative mortality curves for PSM cohorts. (a) For PSM cohort 1 (SDLNM vs. IIIc stage); (b) for PSM cohort 2 (SDLNM vs. other oligometastasis). PSM: propensity score matching; SDLNM: synchronous isolated distant lymph node metastasis; BCSM: breast cancer-specific mortality. Figure S3: receiver operating characteristic (ROC) curves for nomogram predicting 1-, 3-, and 5-year OS prediction of SDLNM patients; (a)–(c) for training cohort; (d)–(f) for validation cohort. AUC: area under roc curve; OS: overall survival; SDLNM: synchronous isolated distant lymph node metastasis.
