# Supplementary material for: Exploring the Prognosis of Breast Cancer with Synchronous Distant Nonregional Lymph Node Metastasis and Establishing a Predictive Model: A Population-Based Study
Source: Biomed Res Int. 2022 Jan 12;2022:5027457. doi: 10.1155/2022/5027457 (PMC8769852; doi:10.1155/2022/5027457)
Supplement: Supplementary Materials — Table S1: characteristics of BC patients with SLDNM or stage TnN3cM0 before and after PSM. Table S2: characteristics of BC patients with SLDNM or other oligometastasis before and after PSM. Figure S1: cumulative mortality curves for patients with diverse stages in different populations ((a) for all included patients; (b) for luminal A subtype; (c) for luminal B subtype; (d) for Her2 + HR- subtype; (e) for TNBC subtype). SDLNM: synchronous isolated distant lymph node metastasis; Her2: human epidermal growth factor receptor type 2; HR: hormone receptor; TNBC: triple-negative breast cancer; BCSM: breast cancer-specific mortality. Figure S2: cumulative mortality curves for PSM cohorts. (a) For PSM cohort 1 (SDLNM vs. IIIc stage); (b) for PSM cohort 2 (SDLNM vs. other oligometastasis). PSM: propensity score matching; SDLNM: synchronous isolated distant lymph node metastasis; BCSM: breast cancer-specific mortality. Figure S3: receiver operating characteristic (ROC) curves for nomogram predicting 1-, 3-, and 5-year OS prediction of SDLNM patients; (a)–(c) for training cohort; (d)–(f) for validation cohort. AUC: area under roc curve; OS: overall survival; SDLNM: synchronous isolated distant lymph node metastasis. [file 5027457.f1.docx]

| **Table S1. Characteristics of BC patients with SLDNM or stage TnN3cM0 before and after PSM** | | | | | | | |
| --- | --- | --- | --- | --- | --- | --- | --- |
| Characteristics | Unmatched cohort | | |  | Matched cohort | | |
|  | Stage | | |  | Stage | | |
|  | SDLNM (%) | TnN3cM0 (%) | p |  | SDLNM (%) | TnN3cM0 (%) | p |
| Age |  |  | 0.086 |  |  |  | 0.569 |
| ≤50y | 218(31.5) | 186(37.4) |  |  | 157(33.8) | 172(37.1) |  |
| 51-65y | 302(43.6) | 205(41.2) |  |  | 205(44.2) | 192(41.4） |  |
| 66-80y | 172(24.9) | 106(21.3) |  |  | 102(22.0) | 100(21.6) |  |
| Race |  |  | 0.128 |  |  |  | 0.239 |
| White | 481(69.5) | 351(70.6) |  |  | 329(70.9) | 326(70.3) |  |
| Black/AI | 135(19.5) | 108(21.7) |  |  | 85(18.3) | 100(21.6) |  |
| Asian | 76(11.0) | 38(7.6) |  |  | 50(10.8) | 38(8.2) |  |
| Marriage |  |  | 0.509 |  |  |  | 0.743 |
| Married | 343(49.6) | 256(51.5) |  |  | 236(50.9) | 241(54.9) |  |
| Single & unknow | 349(50.4) | 241(48.5) |  |  | 228(49.1) | 223(48.1) |  |
| Histology |  |  | 0.817 |  |  |  | 0.245 |
| IDC | 556(80.3) | 402(80.9) |  |  | 367(79.1) | 381(82.1) |  |
| Non-IDC | 136(19.7) | 95(19.1) |  |  | 97(20.9) | 83(17.9) |  |
| SBR grade |  |  | 0.610 |  |  |  | 0.587 |
| Grade 3 | 335(48.4) | 255(51.3) |  |  | 237(51.1) | 242(52.2) |  |
| Grade 1-2 | 184(26.6) | 126(25.4) |  |  | 126(27.2) | 113(24.4) |  |
| Unknow | 173(25.0) | 116(23.3) |  |  | 101(21.8) | 109(23.5) |  |
| T stage |  |  | 0.141 |  |  |  | 0.218 |
| T0-1 | 276(39.9) | 168(33.8) |  |  | 77(16.6) | 68(14.7) |  |
| T2 | 207(29.9) | 163(32.8) |  |  | 145(31.3) | 151(32.5) |  |
| T3 | 107(15.5) | 93(18.7) |  |  | 69(14.9) | 90(19.4) |  |
| T4 | 276(39.9) | 168(33.8) |  |  | 173(37.3) | 155(33.4) |  |
| Subtype |  |  | 0.075 |  |  |  | 0.168 |
| Luminal A | 288(41.6) | 174(35.0) |  |  | 187(40.3) | 164(35.3) |  |
| Luminal B | 127(18.4) | 113(22.7) |  |  | 80(17.2) | 105(22.6) |  |
| Her2+HR- | 99(14.3) | 82(16.5) |  |  | 70(15.1) | 73(15.7) |  |
| TNBC | 178(25.7) | 128(25.8) |  |  | 127(27.4) | 122(26.3) |  |
| Surgery |  |  | <0.001 |  |  |  | 0.491 |
| None | 271(39.2) | 95(19.1) |  |  | 110(23.7) | 95(20.5) |  |
| Lumpectomy | 101(14.6) | 87(17.5) |  |  | 77(16.6) | 79(17.0) |  |
| Mastectomy | 320(46.2) | 315(63.4) |  |  | 277(59.7) | 290(62.5) |  |
| Radiotherapy |  |  | <0.001 |  |  |  | 0.188 |
| Yes | 247(35.7) | 293(59.0) |  |  | 240(51.7) | 260(56.0) |  |
| No | 445(64.3) | 204(41.0) |  |  | 224(48.3) | 204(44.0) |  |
| Chemotherapy |  |  | <0.001 |  |  |  | 0.711 |
| Yes | 578(83.5) | 463(93.2) |  |  | 427(92.0) | 430(92.7) |  |
| No | 114(16.5) | 34(6.8) |  |  | 37(8.0) | 34(7.3) |  |
| AI, American Indian; BC, breast cancer; HR, hormone receptor; | | | | | | | |
| Her2, human epidermal growth factor receptor type 2; IDC, infiltrating duct carcinoma; | | | | | | | |
| PSM, propensity score matching; SBR grade, Scarff-Bloom-Richardson grading system; | | | | | | | |
| SDLNM, synchronous isolated distant lymph node metastasis; TNBC, triple-negative breast cancer. | | | | | | | |

| **Table S2. Characteristics of BC patients with SLDNM or other oligometastasis before and after PSM** | | | | | | | |
| --- | --- | --- | --- | --- | --- | --- | --- |
| Characteristics | Unmatched cohort | | |  | Matched cohort | | |
|  | Stage | | |  | Stage | | |
|  | other oligometastasis (%) | SDLNM (%) | p |  | other oligometastasis (%) | SDLNM (%) | p |
| Age |  |  | 0.100 |  |  |  | 0.538 |
| ≤50y | 2371(28.4) | 218(31.5) |  |  | 215(31.1) | 218(31.5) |  |
| 51-65y | 3631(43.5) | 302(43.6) |  |  | 319(46.1) | 302(43.6) |  |
| 66-80y | 2348(28.1) | 172(24.9) |  |  | 158(22.8) | 172(24.9) |  |
| Race |  |  | <0.001 |  |  |  | 0.031 |
| White | 6364(76.2) | 481(69.5) |  |  | 504(72.8) | 481(69.5) |  |
| Black/AI | 1371(16.4) | 135(19.5) |  |  | 140(20.2) | 135(19.5) |  |
| Asian | 615(7.4) | 76(11.0) |  |  | 48(6.9) | 76(11.0) |  |
| Marriage |  |  | 0.731 |  |  |  | 0.554 |
| Married | 4082(48.9) | 343(49.6) |  |  | 354(51.2) | 343(49.6) |  |
| Single & unknow | 4268(51.1) | 349(50.4) |  |  | 338(48.8) | 349(50.4) |  |
| Histology |  |  | <0.001 |  |  |  | 0.108 |
| IDC | 6056(72.5) | 556(80.3) |  |  | 579(83.7) | 556(80.3) |  |
| Non-IDC | 2294(27.5) | 136(19.7) |  |  | 113(16.3) | 136(19.7) |  |
| SBR grade |  |  | <0.001 |  |  |  | 0.090 |
| Grade 3 | 2462(29.5) | 335(48.4) |  |  | 354(51.2) | 335(48.4) |  |
| Grade 1-2 | 3651(43.7) | 184(26.6) |  |  | 199(28.8) | 184(26.6) |  |
| Unknow | 2237(26.8) | 173(25.0) |  |  | 139(20.1) | 173(25.0) |  |
| T stage |  |  | <0.001 |  |  |  | 0.035 |
| T0-1 | 1247(14.9) | 102(14.7) |  |  | 69(10.0） | 102(14.7) |  |
| T2 | 3266(39.1) | 207(29.9) |  |  | 235(34.0) | 207(299) |  |
| T3 | 1607(19.2) | 107(15.5) |  |  | 117(16.9) | 107(15.5) |  |
| T4 | 2230(26.7) | 276(39.9) |  |  | 271(39.2) | 276(39.9) |  |
| Subtype |  |  | <0.001 |  |  |  | 0.137 |
| Luminal A | 5531(66.2) | 288(41.6) |  |  | 328(47.4) | 288(41.6) |  |
| Luminal B | 1368(16.4) | 127(18.4) |  |  | 120(17.3) | 127(18.4) |  |
| Her2+HR- | 564(6.8) | 99(14.3) |  |  | 79(11.4) | 99(14.3) |  |
| TNBC | 887(10.6) | 178(25.7) |  |  | 165(23.8) | 178(25.7) |  |
| Surgery |  |  | <0.001 |  |  |  | 0.054 |
| None | 4857(58.2) | 271(39.2) |  |  | 301(40.5) | 271(39.2) |  |
| Lumpectomy | 1057(12.7) | 101(14.6) |  |  | 73(10.5) | 101(14.6) |  |
| Mastectomy | 2436(29.2) | 320(46.2) |  |  | 318(46.0) | 320(46.2) |  |
| Radiotherapy |  |  | <0.001 |  |  |  | 0.513 |
| Yes | 2133(25.5) | 247(35.7) |  |  | 237(34.2) | 247(35.7) |  |
| No | 6217(74.5) | 445(64.3) |  |  | 455(65.8) | 445(643) |  |
| Chemotherapy |  |  | <0.001 |  |  |  | 0.773 |
| Yes | 5013(60.0) | 578(83.5) |  |  | 574(82.9) | 578(83.5) |  |
| No | 3337(40.0) | 114(16.5) |  |  | 118(17.1) | 114(16.5) |  |
| AI, American Indian; BC, breast cancer; Her2, human epidermal growth factor receptor type 2; HR, hormone receptor;  IDC, infiltrating duct carcinoma; PSM, propensity score matching; SDLNM, synchronous isolated distant lymph node metastasis; SBR grade, Scarff-Bloom-Richardson grading system; TNBC, triple-negative breast cancer. | | | | | | | |

**
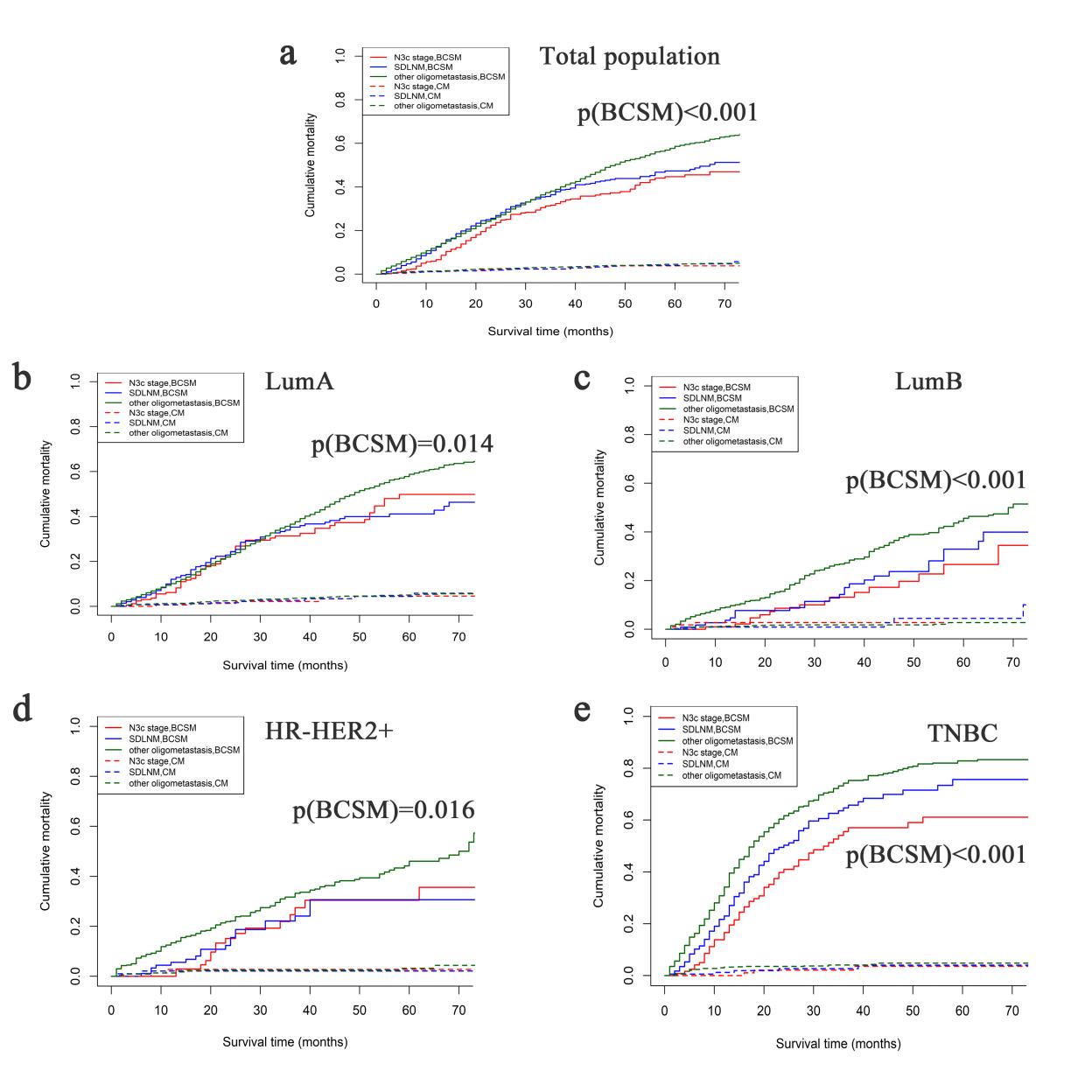
**

**Figure S1.** Cumulative mortality curves for patients with diverse stages in different populations (a, for all included patients; b, for Luminal A subtype; c, for Luminal B subtype; d, for Her2+HR- subtype; e, for TNBC subtype). SDLNM, synchronous isolated distant lymph node metastasis; Her2, human epidermal growth factor receptor type 2; HR, hormone receptor; TNBC, triple-negative breast cancer; BCSM, breast cancer-specific mortality

**
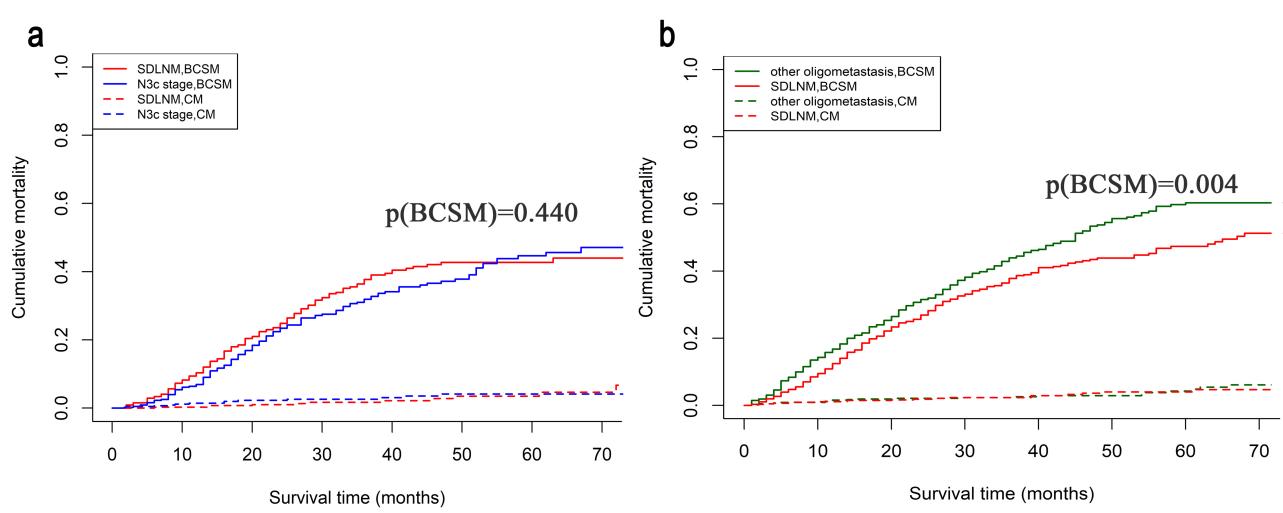
**

**Figure S2.** Cumulative mortality curves for PSM cohorts. (a) for PSM cohort 1 (SDLNM vs IIIc stage); (b) for PSM cohort 2 (SDLNM vs other oligometastasis). PSM, propensity score matching; SDLNM, synchronous isolated distant lymph node metastasis; BCSM, breast cancer-specific mortality

**
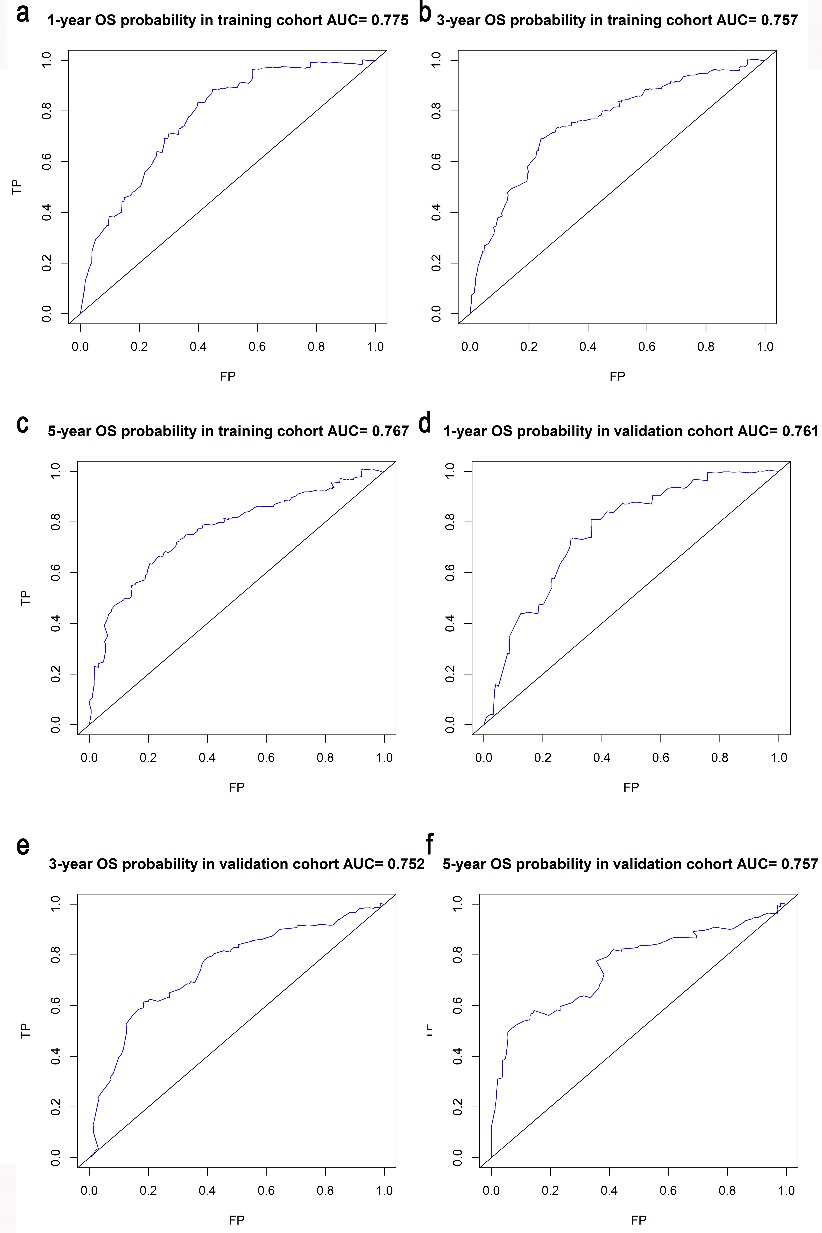
**

**Figure S3.** Receiver Operating Characteristic (ROC) curves for nomogram predicting 1-, 3- and 5-year OS prediction of SDLNM patients; (a-c) for training cohort; (d-f) for validation cohort

AUC, area under roc curve; OS, overall survival; SDLNM, synchronous isolated distant lymph node metastasis
